# Turning up the Heat: Increasing Temperature and Coral Bleaching at the High Latitude Coral Reefs of the Houtman Abrolhos Islands

**DOI:** 10.1371/journal.pone.0043878

**Published:** 2012-08-29

**Authors:** David A. Abdo, Lynda M. Bellchambers, Scott N. Evans

**Affiliations:** Marine Ecology and Monitoring Section, Biodiversity and Biosecurity Branch, Department of Fisheries, Government of Western Australia, Hillarys, Western Australia, Australia; Ohio State University, United States of America

## Abstract

**Background:**

Coral reefs face increasing pressures particularly when on the edge of their distributions. The Houtman Abrolhos Islands (Abrolhos) are the southernmost coral reef system in the Indian Ocean, and one of the highest latitude reefs in the world. These reefs have a unique mix of tropical and temperate marine fauna and flora and support 184 species of coral, dominated by *Acropora* species. A significant La Niña event during 2011 produced anomalous conditions of increased temperature along the whole Western Australian coastline, producing the first-recorded widespread bleaching of corals at the Abrolhos.

**Methodology/ Principal Findings:**

We examined long term trends in the marine climate at the Abrolhos using historical sea surface temperature data (HadISST data set) from 1900–2011. In addition *in situ* water temperature data for the Abrolhos (from data loggers installed in 2008, across four island groups) were used to determine temperature exposure profiles. Coupled with the results of coral cover surveys conducted annually since 2007; we calculated bleaching thresholds for monitoring sites across the four Abrolhos groups.

**Conclusions/ Significance:**

*In situ* temperature data revealed maximum daily water temperatures reached 29.54°C in March 2011 which is 4.2°C above mean maximum daily temperatures (2008–2010). The level of bleaching varied across sites with an average of ∼12% of corals bleached. Mortality was high, with a mean ∼50% following the 2011 bleaching event. Prior to 2011, summer temperatures reached a mean (across all monitoring sites) of 25.1°C for 2.5 days. However, in 2011 temperatures reached a mean of 28.1°C for 3.3 days. Longer term trends (1900–2011) showed mean annual sea surface temperatures increase by 0.01°C per annum. Long-term temperature data along with short-term peaks in 2011, outline the potential for corals to be exposed to more frequent bleaching risk with consequences for this high latitude coral reef system at the edge of its distribution.

## Introduction

Coral reefs are usually associated with warm, shallow waters within the tropics [1], [2], as coral growth is generally limited to areas where water temperatures remain above 18°C [1], [2], [3], [4]. Coral reef ecosystems are ecologically, economically and culturally significant. They are recognized as ‘hot-spots’ of biodiversity, as well as important resources for fisheries and tourism industries [5], [6], [7]. A multitude of ecosystem services are attached to coral reefs with conservative estimates valuing coral reefs globally between $172–375 billion (USD) per year [8]. With significant links to society, coral reefs attract an appreciable research and management focus, particularly when the levels of disturbance that threaten their persistence are considered.

Along with cyclical natural impacts (e.g. cyclones) coral reefs face anthropogenic pressures (e.g. climate change, coastal development, fishing and tourism) [7]. Mounting evidence suggests that coral reefs are unlikely to cope with the multitude of stresses (e.g. predation by the coral-eating crown-of-thorns starfish, sedimentation impacts, over-fishing and destructive fishing practices, eutrophication, pollution, diseases, and global warming) that are increasingly affecting them [7], [9], [10]. As a result it is estimated that 19% of the world's coral reefs have already been lost, with a further third facing extinction [8].

Global climate change is a particular threat to coral reefs and corals specifically because many coral species exist close to their thermal maxima, with slight or sustained temperature increase leading to bleaching [7], [11], [12]. It is now well established that increased temperature plays a significant role in disrupting the symbiosis between coral hosts and zooxathellae leading to coral bleaching [13], [14], although localized bleaching can have numerous causes [15]. This phenomenon has been reported around the globe and for numerous species of corals [8], [11], [16], as well as other coral reef invertebrates such as sponges [17] and giant clams [18]. This is a result of coral reefs requiring distinct environmental conditions [2], [11], with exceedances of these conditions leading to physiological stress and mortality [11]. If the effect is short lived, the corals may recover, otherwise it can lead to disease and death in the affected corals [8].

The majority of the world's major coral reefs have been affected by bleaching related to thermal anomalies [13], [19], [20], however, high latitude coral reefs (>26° latitude) have been largely unaffected by thermal bleaching [21], [22], [23], [24]. It has been suggested this is a result of the relatively stable seawater temperature regime in which they occur and the documented climate-induced pole ward shift (over geological and ecological temporal scales) of coral reef taxa [16], [25], [26], [27], [28]. High latitude coral reefs are likely to be important refugia from the impacts of climate change [23], [29]. However, questions remain around the response of these reefs to increasing temperatures and ocean acidification, and how the interplay of different stresses may affect them [30]. At high latitudes, coral distribution and abundance is influenced by a variety of factors, e.g. lower water temperature [2] and increased competition with macroalgae [1], [3], that can result in reduced growth rates, reproduction and viability [31], [32].

**Figure 1:**
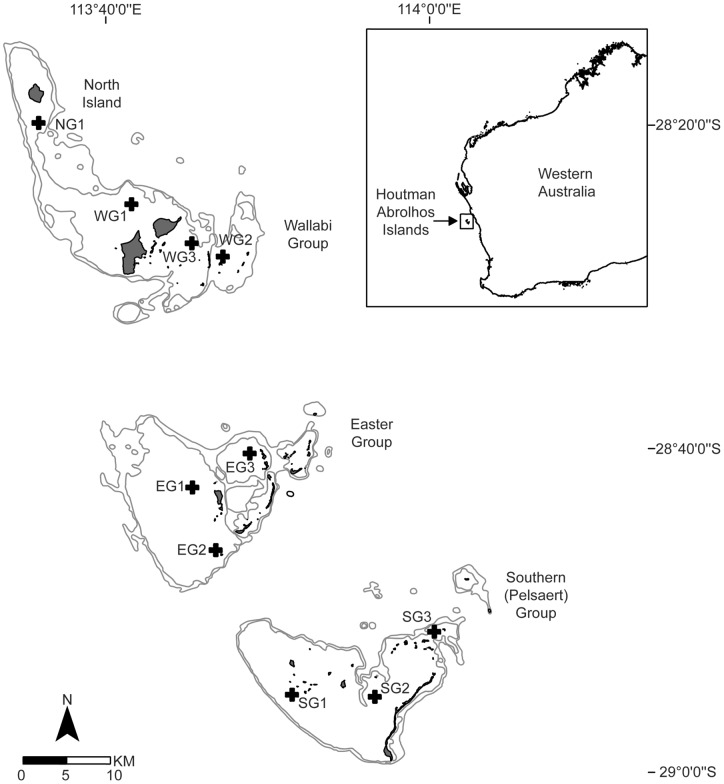
Location of the Abrolhos relative to mainland Australia and the location of the long-term monitoring sites where surveys of benthic cover, collection of *in situ* water temperature and water motion data occurs.

The Houtman Abrolhos Islands (Abrolhos) are one of the highest latitude coral reef systems in the world and the southernmost in the Indian Ocean [33], [34]. They are located on the edge of the Western Australian continental shelf between 28°16′S and 29°00′S [34], [35], in the pathway of the warm poleward flowing Leeuwin Current [36] (Figure 1). As a temperate/tropical mixture zone the Abrolhos has an exceptional range of marine diversity including coral fauna, with 184 species from 42 genera [35]. This is primarily due to the influence of the Leeuwin Current transporting tropical marine fauna southwards from northern Australia [37], with tropical species co-existing with temperate species including habitat structuring alga [38]. The Abrolhos also supports significant commercial fisheries (e.g. ∼19% of annual Western Rock Lobster fishery worth ∼$37 million (AUD) is taken from the Abrolhos region [39]), with ever increasing tourism and recreational fishing activities [40].

Recent research has shown a significant shift in the West Australian marine climate, with warmer water reaching further south [33]. Coral reef taxa (over geological time scales) have responded to a warming climate along the WA coast [26], [41]. This suggests that the Abrolhos may have the potential to act as a coral refuge [29], where corals may survive better under a warming marine climate. Thermal thresholds for corals can be described being generally 1–2°C above long term summer maxima [8] and have been also determined experimentally for several species and areas [42]. However, it is not clear how these limits apply *in situ* where several processes (e.g. wind and wave action) can work synergistically to alter the effects of thermal stress [43]. Understanding the thresholds of coral reefs will be key for developing adaptive management strategies [19], [43], such as marine spatial planning which will increase the potential for coral reefs to cope with climate change by mitigating non-climate-related stressors [7], [8], [19]. These localized strategies can only be beneficial alongside strong policy with respect to climate change [7], [8], [19].

Here, we report on the first recorded large scale bleaching event at the Abrolhos that occurred during the austral summer of 2011. We examine long-term sea surface temperature trends at the Abrolhos to understand the patterns in thermal stress; and calculate time-temperature thresholds for the Abrolhos coral reefs from *in situ* temperature data to understand the potential implications for reefs under a changing climate.

## Results

### Sea surface temperature trends

**Figure 2:**
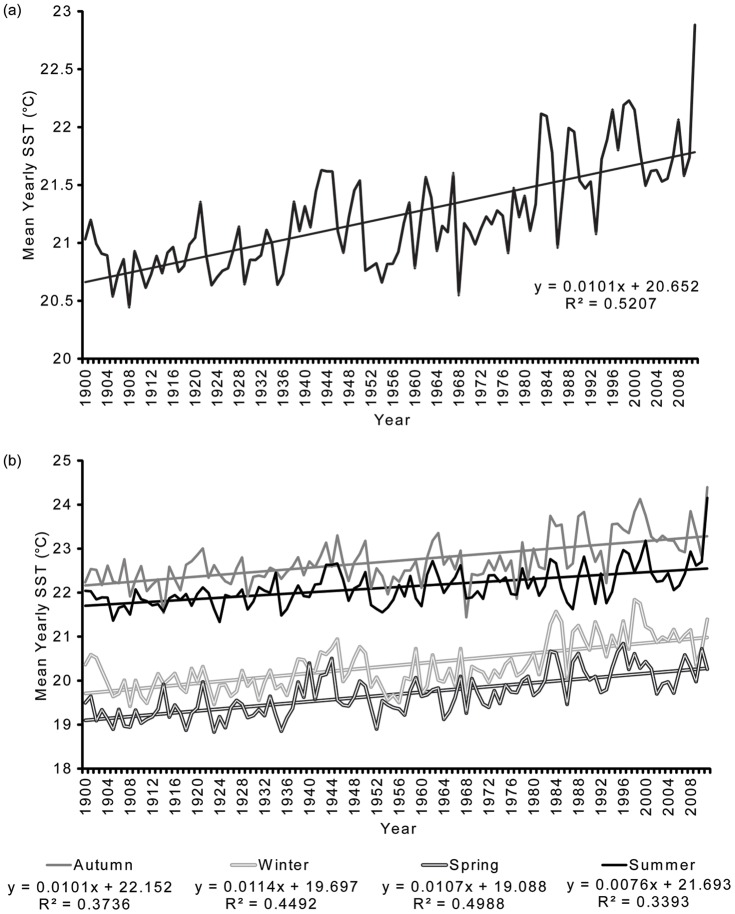
Trend in mean yearly SST (a); and seasonal mean yearly SST (b) at the Abrolhos Islands using the (based on a two degree block – 27.5–29.5°S, 113.5°E) using HadISST data. Summer is defined as being Dec-Feb, autumn as Mar-May, winter as Jun-Aug and spring as Sep-Nov.

The HadISST dataset had a significant positive relationship with mean monthly *in situ* water temperature (WT) data recorded across 10 monitoring sites at the Abrolhos (R^2^ = 0.8917, F _(1,42)_  = 345.9, P<0.001). Based on the HadISST dataset, the waters surrounding the Abrolhos have experienced a significant increase in mean yearly SST since 1900 (R^2^ = 0.521, F_ (1,110)_  = 119.5, P<0.001) at the rate of 0.010°C yr^−1^ (Figure 2). The rate of mean yearly SST increase since 1900 was largest in the austral winter (Jun-Aug) and spring (Sep-Nov) at 0.012°C yr^−1^ and 0.011°C yr^−1^ respectively (Figure 2). In autumn (Mar-May) and summer (Dec-Feb), the rate of increase was 0.010°C yr^−1^ and 0.007°C yr^−1^ respectively (Figure 2). These differences among seasons were not significantly different (Table 1).

**Table 1:** ANCOVA results testing homogeneity of slopes of mean yearly SST (based on HadISST data, from January 1900– September 2011) rate of change among seasons (Summer, Autumn, Winter, Spring). Significant interaction would indicate heterogeneity of slopes.

	Df	Sum Sq	Mean Sq	F value	Pr(>F)
Year	1	46.39	46.392	312.2251	**<0.001**
Season	3	692.07	230.689	1552.5721	**<0.001**
Year:Season	3	0.96	0.320	2.1509	0.09315
Residuals	440	65.38	0.149		

Significant result at α = 0.05 indicated in bold.

### In situ water temperature regime

During 2011, mean yearly seawater temperature at the Abrolhos was 22.91°C (±0.10°C). This is approximately 1°C above the mean yearly seawater temperature (21.94°C ±0.19°C) over the 2008-010 period, and approximately 1.5°C above the historical climatological yearly SST average for the Abrolhos (21.34°C ±0.07°C; based on HadISST data for the period 1961–990, see [44]). The daily mean seawater temperature at the Abrolhos was significantly different between the monitoring sites over the 6 month period between December 2010 to May 2011(F_(7,1455)_  = 10.05, P<0.001). The hottest period was the last week of February 2011 to mid-March 2011. The hottest day for most sites occurred on the 4^th^ of March. The hottest site was SG1 in the first week of March with a maximum daily temperature of 29.54°C (Table 2). The coolest site was SG3 which had a maximum daily temperature of 27.29°C during the same period (Table 2). The 2011 temperatures were at least 4.7°C hotter than the historical average for period between December and May (based on HadISST data from 1961–1990), and 4.3°C hotter than the same period from 2008–2010 (22.99°C ±0.21°C) (Table 2).

**Table 2:** Summary statistics from the *in situ* seawater temperature data loggers for monitoring sites for 2008–010 (Dec-May) and for 2010/2011 (Dec-May).

	2008−2010			2011		
Site	Min	Max	Mean (± SE)	Min	Max	Mean (± SE)
SG1^2008–2009^	20.90	25.84	23.66 (0.09)	21.14	29.54	25.26 (0.17)
SG2	20.98	25.68	23.16 (0.04)	21.25	27.98	24.57 (0.12)
SG3^2010 only^	21.17	25.00	22.95 (0.04)	22.41	27.29	24.80 (0.09)
EG1	20.31	25.58	23.15 (0.05)	21.47	28.2	24.67 (0.11)
EG2	20.82	25.78	23.18 (0.05)	21.50	27.95	24.56 (0.11)
EG3	19.75	24.62	22.30 (0.11)	21.29	27.27	24.10 (0.11)
WG1	20.73	25.36	23.20 (0.04)	22.57	28.79	25.19 (0.11)
WG2^2010 only^	20.73	24.78	22.84 (0.08)	22.62	28.55	25.12 (0.11)
WG3^2010 only^				23.24	28.63	25.39 (0.14)
NI1	20.37	26.02	23.19 (0.05)	21.51	28.54	24.79 (0.12)
Pooled Sites	20.64 (0.14)	25.41 (0.17)	23.07 (0.12)	21.90 (0.23)	28.27 (0.22)	24.85 (0.13)

**Figure 3:**
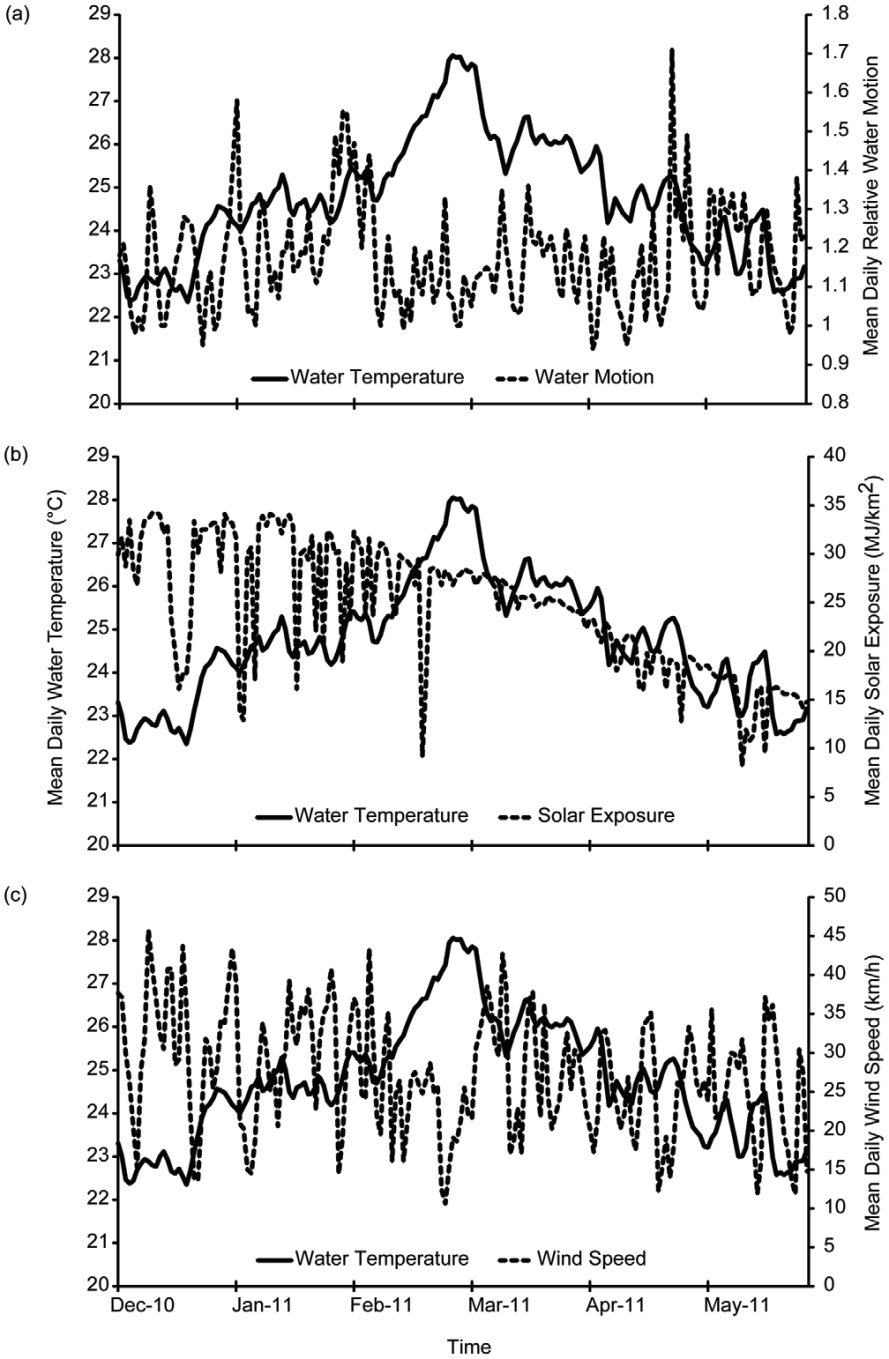
Mean daily water temperature and (a) water motion, (b) solar exposure and (c) wind speed for the Abrolhos Islands between December 2010 and May 2011 .

**Figure 4:**
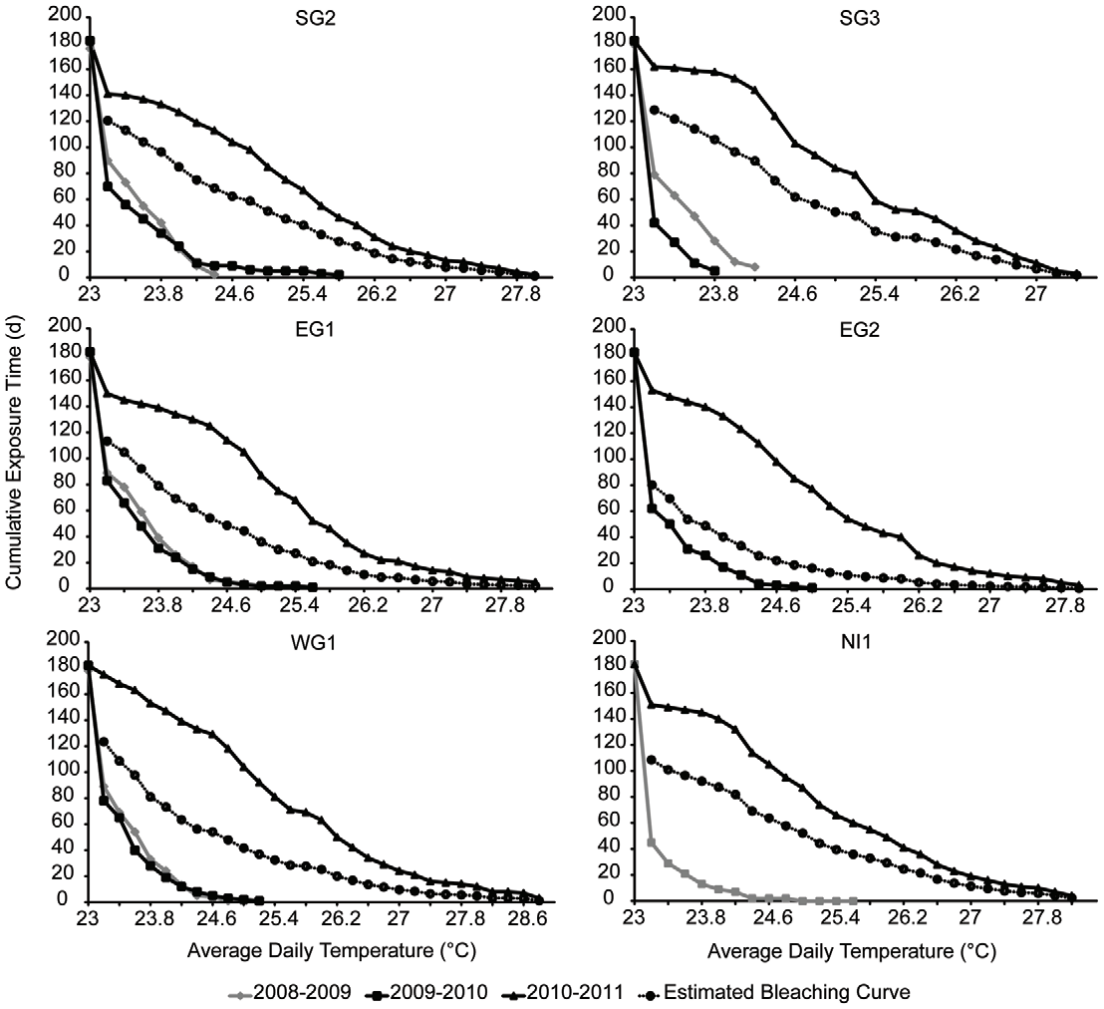
Thermal exposure curves for the Abrolhos monitoring sites. Note y axes have different scales. SG1, WG2, WG3 site did not have complete data for threshold curves to be determined.

Increased mean daily seawater temperature at the Abrolhos did correspond to decreased water motion, solar exposure and mean wind speed (Figure 3). This was most apparent during the hottest months of February and March 2011 where seawater temperatures increased during periods of lower water motion and wind strength (Figure 3). Regression analysis revealed that local environmental conditions (i.e. daily solar exposure, wind speed and water motion) accounted for 8.9% of the variation in daily seawater temperatures (R^2^ = 0.0896, F _(3,178)_  = 5.842, P<0.001) (Figure 4). Both daily solar exposure (t = 3.624, P<0.001) and daily wind speed (t = −2.829, P = 0.005) had a significant relationship with daily seawater temperatures. Water motion (t = 0.423, P = 0.673) did not have a significant influence on the variability in mean daily seawater temperature at the Abrolhos during the summer 2011 bleaching period.

### Coral bleaching intensity and mortality

Across all sites the mean level of bleaching was 12.16% (±4.39%) (Table 3). Bleaching ranged from a moderate level of 1.95% at SG3 to a very high level of 42.46% at EG2 (Table 3). Mortality varied among sites, with a mean mortality of 48.57%. Site SG2 had an average of 62.10% (±4.56%) ‘healthy’ hard coral prior to 2011, but has had almost a total loss of coral cover (99.85%) (Table 3). Prior to 2011, the mean level of bleaching at any of the monitoring sites did not exceed 1% (0.64% ±0.14%) (i.e. bleaching at site EG1).

**Table 3:** Mean percentage cover of bleached hard coral BL), corresponding bleaching category (BC), and mean percentage mortality (M) for the coral monitoring sites at the Abrolhos 2011.

Site	BL	BC	M
SG1	6.10 (1.72)	4	
SG2			99.85 (0.15)
SG3	1.95 (0.50)	4	9.30 (3.34)
EG1	10.21 (3.02)	3	37.02 (6.58)
EG2	42.46 (21.11)	2	58.03 (6.01)
EG3	9.55 (1.56)	4	54.98 (1.91)
WG1	23.55 (6.57)	3	32.27 (10.43)
WG2	4.39 (1.02)	4	
WG3	10.16 (1.95)	3	
NI1	1.09 (0.57)	4	
Abrolhos Mean	12.16 (4.39)		48.57 (1.51)

Numbers in brackets represent one standard error. Bleaching categories are: 1 (extreme, >60% bleached), 2 (very high, 30–60% bleached), 3 (high, 10–30% bleached), 4(moderate, 1–10% bleached), and 5 (no or low bleaching, <1%). Note surveys in February 2011 at site SG2 could not be completed due to low visibility (see discussion). Surveys in May 2011 were not completed at sites SG1, WG2, WG3 and NI1.

### Bleaching thresholds

**Figure 5:**
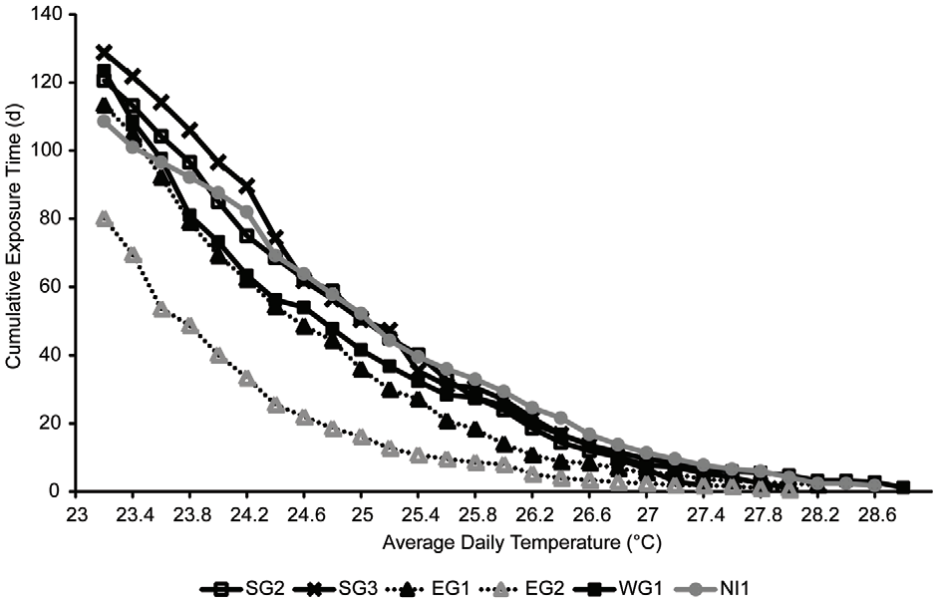
Comparison of estimated bleaching curves among sites at the Abrolhos, illustrating variability in thresholds for site EG2.

Thermal tolerance curves were estimated for each monitoring site (excluding EG3, SG1, WG1 and WG3 due to incomplete datasets) for the 2008/2009, 2009/2010 and 2010/2011 periods (Figure 4; Table 4). For example at EG1, maximum mean daily temperatures reached 25.6°C for 1 day during 2009/2010 while during 2010/2011, temperatures reached 28.2°C for 5 days (Figure 4; Table 4). Mean daily temperatures at EG1 were over 25.6°C for 46 days in 2010/2011 (Table 4). In 2010/2011 SG3 experienced 27.4°C for 3 days, with seawater temperatures being greater than the previously highest recorded mean daily temperature exposure for 136 days (during 2008/2009 when the corals experienced 24.2°C for 8 days) (Table 4). Comparison of bleaching curves revealed that most sites (with available data) had relatively similar bleaching threshold curves, except for sites SG2 and EG2 which had much lower thresholds (Figure 5).

**Table 4:** ummary of cumulative exposure of monitoring sites across the Abrolhos for the hottest year when bleaching occurred, and for the warmest previous year on record. Difference in exposure is the number of days the mean daily temperature was above the previous warmest year with no bleaching.

	Hottest Year		Difference in Exposure (d)	Previous Warmest Year		
Site	Temp (°C)	Cumulative Exposure (d)		Temp (°C)	Cumulative Exposure (d)	Year
SG1	-	-	-	-	-	-
SG2	28.0	2	44	25.8	2	2009/2010
SG3	27.4	3	136	24.2	8	2008/2009
EG1	28.2	5	51	25.6	1	2009/2010
EG2	28.0	3	76	25	1	2009/2010
EG3	27.4	4	-	-	-	-
WG1	28.8	3	91	25.2	1	2009/2010
WG2	-	-	-	-	-	-
WG3	-	-	-	-	-	-
NI1	28.6	3	93	24.8	2	2009/2010
Abrolhos Average	28.1 (±0.20)	3.3 (±0.36)	81.8 (±13.6)	25.1 (±0.24)	2.5 (±1.12)	

### Discussion

**Figure 6:**
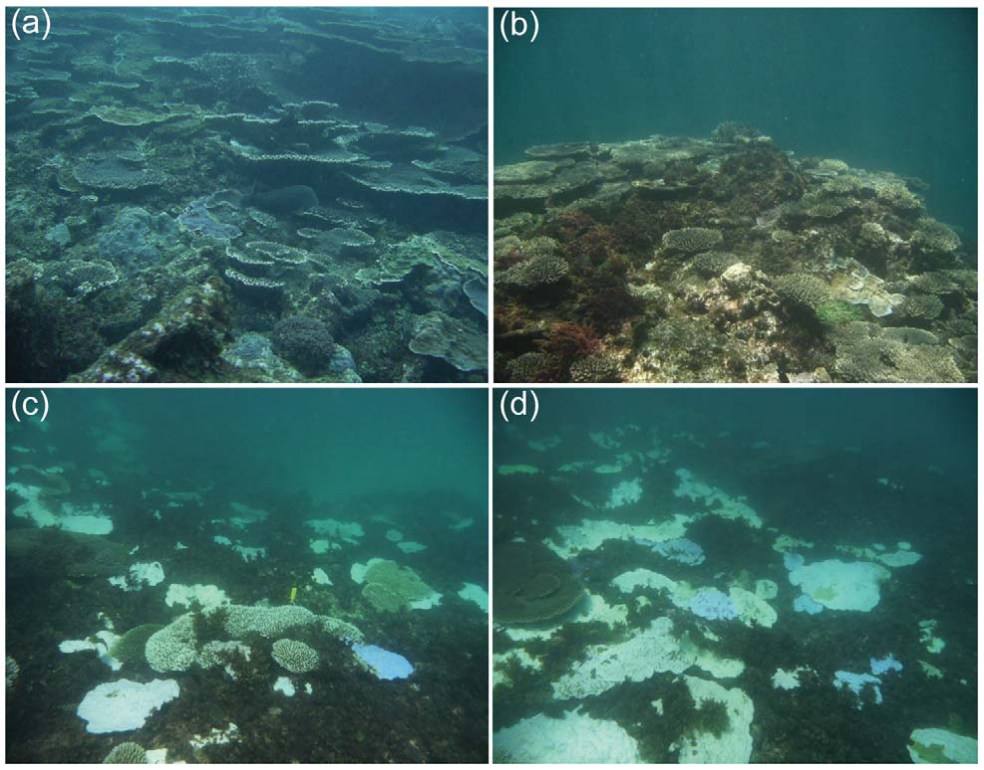
Images depicting the coral reefs of the Abrolhos prior to the 2011 bleaching event at site EG1 (a–b), and during the 2011 bleaching event at site EG2 (c–d).

Despite previous widespread bleaching throughout the Indian Ocean [21], [45] and long-term increasing trend in seawater temperatures at the Abrolhos [shown here and 33,46,47], wide spread bleaching was recorded for the first time at the Abrolhos in 2011 [34]. Bleaching at the Abrolhos was on average high (∼12%) however the level of bleaching varied significantly between sites ranging from moderate through to total loss of coral (Figure 6). Although, this is an underestimate of the 2011 bleaching event due to the timing of our surveys with surveys. Surveys in April 2011 by an autonomous underwater vehicle (AUV) of some deeper reefs (∼20 m) near the Easter Group recorded up to 20% bleaching [48]. The overall level of bleaching recorded is comparable to that seen in other tropical regions of the world [49], [50], [51] and high latitude coral reefs [21], [23]. In 2010 extensive bleaching was recorded at Lord Howe Island (the highest latitude coral reef in the world) with some sites suffering up to 90% bleaching, and up to 25% mortality at some monitoring sites [22]. Here we recorded the total loss of coral cover at one monitoring site (mean of ∼50%), and subsequent visits to the Abrolhos have observed further losses in coral cover (e.g. SG1 with 100% loss of coral; *S. Evans pers comm*). The loss of coral cover at the Abrolhos may be compounded by the effects of disease and/or predation, which can result after long periods of stress (like disturbance from bleaching) [52], [53], [54]. In the Caribbean, widespread disease outbreaks were recorded following the 2005 bleaching with coral cover declining by a further 61% over the following two years [54]. The true impact of the 2011 bleaching event at the Abrolhos is only likely to be seen after subsequent monitoring surveys are completed in 2012 and beyond.

It is clear that the primary driving force of the thermal bleaching at the Abrolhos was at a regional scale (>1000 km's) due to the anomalous La Nińa event and associated record strength Leeuwin Current [55], with little (∼9%) of the variability in mean sea temperatures being explained by local conditions (i.e. wind, water motion or solar exposure). However, within the Abrolhos we observed significant differences in the mean seawater temperature as well as differences in bleaching level between monitoring sites, this variability in bleaching intensity may be due to several factors. Firstly, the timing of our surveys (i.e. first survey being undertaken before the temperature maxima) would have underestimated the scale of the 2011 bleaching event as recent observations of SG1 have revealed a total loss of coral at this site (*S. Evans pers. comm*).

There is also anecdotal evidence that the loss of coral cover at several sites (e.g. EG2) may have been compounded by increased turbidity. The Abrolhos turbidity was likely the result of an algal bloom, as local weather conditions were not conducive to increased suspension of sediments (reported here). Further, experiments on *Acropora intermedia* showed that high suspended sediment loads (i.e. turbidity) decreased mortality levels when associated with increased thermal stress [56]. The observed algal bloom is likely to have reduced oxygen levels within the water column thereby increasing coral stress and mortality. This hypothesis is supported by reports of ‘fish kills’ at the Abrolhos during the same period in 2011 [55].

The *in situ* data collected has allowed us to determine thermal tolerance curves and bleaching threshold curves for the corals of the Abrolhos. The thresholds for the Abrolhos are considerably lower than those reported for other reefs. While Abrolhos corals reached similar maximum threshold temperatures (on average 28.8°C for 1 day), compared to tropical reefs like Heron Island (∼23.4°S latitude) which reached 29.3°C for ∼1 day [57]. The duration of exposure at higher temperatures was much lower than that seen in tropical reefs and other high latitude reefs. On average Abrolhos corals experienced temperatures >27.6°C ∼4 day, whereas at Heron Island, corals could similar temperatures for ∼25 days [57]. At Sodwana Bay (∼27°S latitude), corals experienced durations of ∼35 days at temperatures >27.6°C [21]. The bleaching thresholds curves of the Abrolhos represent among the lowest reported, suggesting a high thermal sensitivity for Abrolhos corals. Further, the predicted curves help define the relationship between temperature and exposure time for a range of locations within the Abrolhos Islands. The bleaching thresholds were relatively consistent between sites at the Abrolhos except for one monitoring sites (EG2). The estimated bleaching curves for EG2 may have been influenced by algal blooms that occurred during the bleaching period overweighting the influence of thermal stress at this site reducing the thermal tolerance threshold. The collection of additional *in situ* data may clarify the factors influencing the thermal thresholds of the Abrolhos coral reefs. Nevertheless, the bleaching curves provide an assessment of the vulnerability of the Abrolhos to climate change. Acroporid species are the dominant species at the Abrolhos [35] and are also among the most sensitive corals [58]. The bleaching curves defined here represent the thresholds for the most important structural element of the Abrolhos' reefs. This is important for effective management of the Abrolhos reef system allowing long term planning (such as strategies to reduce localized anthropogenic disturbances) to ensure the maintenance of the ecological, economic and social resources that the Abrolhos provides.

Globally coral reefs are increasingly under threat [11] with a steady increase in the number of reefs reaching critical ecological and social thresholds [8], [11]. The long-term trend of increasing SST for the Abrolhos [shown here and by 33,46,47], suggests that the Abrolhos reefs may be increasingly subjected to thermal bleaching. However, recent work suggests that reefs at the edge of their distribution (including the Abrolhos) have adapted to climate induced increases in temperature [26], [41]. Cooper et al. (2011) revealed that increases in SST anomalies since the early 1900’s resulted in increased calcification rates for massive *Porites* colonies. Further, it is well documented that the Leeuwin Current dominates the marine climate of Western Australia and is responsible for the dispersal of marine organisms from the tropical north southwards [23], [27], [29]. This raises the question as to whether the supply of ‘heat tolerant’ recruits from the tropical north to the Abrolhos will be sufficient to overcome the warming marine climate at the Abrolhos, and provide a level of resistance to climate change.

Most corals have broad geographic distributions and are capable of large scale (>1000 km's) dispersal at least occasionally [59]. This is particularly relevant to corals which broadcast spawn (undergo fertilization and larval development within the water column) versus those which brood their larvae following internal fertilization [59]. Under future climate scenarios (for the west coast of Australia), a predicted decrease (15%) in Leeuwin Current transport is expected towards 2060 [60]. Coupled with recent work that has shown that larval pre-competency periods are reduced by elevated temperatures, potentially reducing dispersal ranges [61]. The likelihood of suitable long-distance dispersal (especially for broadcast spawners) from the tropical north, to replenish Abrolhos coral populations, may be even more limited. Further, localized depletion of corals could decrease the viability of the Abrolhos coral populations by reducing the supply of locally produced larvae. A study of the population structure of *Pocillopora damicornis* at the Abrolhos indicates that *P. damicornis* populations are primarily self-seeding [59]. Noreen et al. (2009) also found that gene flow from tropical populations of brooding *Seriatopora hystrix* to southern high latitude populations along the East coast of Australia would be insufficient (over ecological time-scales) to allow recovery of the reefs [62]. This suggests that if temperature pulses like that observed here in 2011 increase in frequency, the capacity of natal sources of new recruits may decrease, thus reduce the potential stability of the Abrolhos coral populations [63]. This study is the first to document thermal thresholds and the spatial distribution of bleaching at the Abrolhos Islands. It provides a valuable first step in understanding the impacts of future disturbance events, which is critical to assess the resilience of high latitude coral communities, like the Abrolhos, under the predicted increasing cycle of disturbance [11], [12], [16]. A better understanding of the processes driving disturbance and recovery will inform adaptive management strategies to increase the resilience of coral communities to climate change.

## Materials and Methods

### Long-term Sea Surface temperature trends

Monthly sea surface temperature (SST) data between January 1900 to September 2011 were extracted from the HadISST dataset for two 1° latitude by 1°longitude boxes covering the Abrolhos [64]. SST data were averaged for the two boxes (covering an area from 27.5–29.5°S, 113.5–114.5°E) to give an estimate of the long-term SST conditions at the Abrolhos. To confirm the suitability of the HadISST dataset for understanding long-term trends at the Abrolhos, linear regression analysis was performed between the mean monthly *in situ* temperature data averaged across all sites (and the monthly HadISST data between February 2008 and September 2011). *In situ* seawater temperature data has been collected on an hourly basis since February 2008 at long term monitoring sites throughout the Abrolhos (further details of *in situ* water temperature data loggers below).

Long-term trends in mean yearly and seasonal SST trends were examined through linear regression. Differences among the rate of change between seasons (i.e. the slopes of each season's linear regression) were examined using a one-way analysis of co-variance (ANCOVA). Data were checked for normality and homogeneity of variance [65].All analyses were performed in RStudio v2.14 using the ‘car’ package.

### In situ water temperature regime

Water temperature data was recorded hourly *in situ* at ten sites spread across the four island groups of the Abrolhos, using the Hobo Pendant temperature loggers (http://www.onsetcomp.com/products/data-loggers/ua-001-64; accessed 24/07/2012) (Figure 1). Water temperature has been recorded since February 2008 for all sites except for EG3, WG2 and WG3 which began in 2010). Differences in mean daily seawater temperature among sites between December 2010 and May 2011 were examined using one-way analysis of variance (ANOVA) (Site with 8 levels, fixed factor). Note SG1 and WG3 were excluded as they did not have a complete data set for the period. Data were checked for normality and homogeneity of variance [65].

To determine the environmental conditions associated with the recorded mass bleaching at the Abrolhos in 2011, multiple linear regression analysis was performed. Daily mean wind speed, daily mean solar exposure (MJ/km^2^) and daily mean relative water motion (averaged across all monitoring sites) were examined for any relationship with *in situ* seawater temperature data (averaged across all monitoring sites)during the December 2010 to May 2011 period. Data were checked for normality and homogeneity of variance [65].

Mean daily water motion was calculated from measurements of relative water motion recorded (at 20 min intervals) at each monitoring site using purpose designed data loggers [66]. Water motion loggers were deployed at the same time and location as the water temperature loggers. Wind data was obtained from the Bureau of Meteorology (BOM) Abrolhos weather station (North Island −28°17′S, 113°35′E) for the 2010/2011 austral summer (December to May) it was assumed that the data was representative of the whole of the Abrolhos. Mean daily wind speed and maximum daily wind speed were calculated. Solar exposure data were obtained from BOM for Geraldton (the station nearest to the Abrolhos). We used the solar exposure data as a proxy for insolation (inversely cloudiness and/or shading) at the water surface.

### Coral cover and bleaching intensity

Long term monitoring of benthic cover of major taxa at the Abrolhos has been recorded since 2007 (in February of each year) from seven reef monitoring sites with additional sites added in 2010 (EG3, WG2 and WG3) (Figure 1). This research was performed in line with the Department of Fisheries exception permit 1094 issued under the Fish Resources Management Act 1994. Reefs are surveyed along 3×100 m transects at each site using digital video. Subsequent analyses of benthic cover is determined by a project specific adapted point count methodology [67], [68], [69], proving more suitable to the unique complexity of algal and coral mix of the Abrolhos. A total of 80 video frames are sampled randomly within approximately 10 min of transect footage (i.e. 1 frame every ∼8 sec) within the TransectMeasure program (www.seagis.com.au; accessed 24/07/2012). Within the analysis software 20 equal squares are overlaid onto each video frame (i.e. twenty 5% squares). The dominant benthic cover was then qualitatively identified for each 5% square per frame. Monitoring surveys occurred in February 2011 prior to the temperature maxima in March 2011. Follow up surveys occurred in May 2011 (except at sites SG3, WG2/WG3 and NI1). The relative level of bleaching at each site was assessed using the following equation:
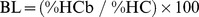
Where, BL is the bleaching level of a site; %HCb is the mean cover bleached hard coral in February 2011 and %HC is the mean cover of ‘healthy’ hard coral in February 2010. It should be noted that the level of bleaching will be an under estimate due to the timing of the February 2011 surveys with respect to the March 2011 temperature maxima. After determining the level of bleaching, sites were then ranked into five categories of bleaching severity [43]; with category 1 being extreme (>60% bleached), 2 (very high, 30–60% bleached), 3 (high, 10 to 30% bleached), 4 (moderate, 1–10% bleached), and 5 (no or low bleaching, <1%). These rankings were then used in understanding the bleaching thresholds at the monitoring sites.

For sites which were resurveyed in May 2011, the level of mortality was calculated by the following equation:



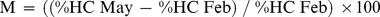
Where, M is the level of mortality at each site; %HC May is the mean percentage of ‘healthy’ hard coral cover in May 2011; %HC Feb is the mean percentage of ‘healthy’ hard coral cover in Feb 2010.

### Bleaching thresholds

Using the *in situ* temperature data from the Abrolhos (collected since 2008) and following the methodology of Berkelmans (2002); the cumulative time (in days) at each temperature (0.2°C increments) was calculated for the period December to May (which covers the austral summer) for each year of available data at each location. The resultant time-temperature curves represent the tail end of the cumulative frequency distributions of water temperatures for each year [43]. A bleaching threshold curve for each site was then interpolated through the average number of days at each 0.2°C increment of the warmest, non-bleaching year and the coolest bleaching year accounting for the bleaching severity recorded at each site [43]. This methodology is considered more robust than thresholds estimated as accumulated heat stress (e.g. degree-days thresholds) due to the time-integrated thresholds not falsely assuming a linear relationship between exposure time and temperature [43], [70].
